# Mental health and quality of life of migrants in transit through the Darién gap: A cross-sectional assessment in Panama

**DOI:** 10.1016/j.jmh.2026.100405

**Published:** 2026-03-20

**Authors:** Justo Pinzón-Espinosa, Jennifer Toller Erausquin, Eugenia Flores Millender, Tomás Rincón, Gonzalo Cabezas Talavero, Carlos Franco-Paredes, Narcis Cardoner, Casey D. Xavier Hall, Frankie Y. Wong, Liying Wang, Carmen Alvarez, Amanda Gabster

**Affiliations:** a Department of Psychiatry, Amsterdam University Medical Center, Amsterdam, the Netherlands; b Department of Clinical Psychiatry, School of Medicine, University of Panamá, Panamá City, Panamá; c Center of Population Sciences for Health Empowerment, Florida State University, Tallahassee, FL, USA; d Department of Public Health Education, University of North Carolina at Greensboro, Greensboro, NC, United States; e INDICASAT-AIP, Panama City, Panama; f College of Social Work, Florida State University, Tallahassee, FL, USA; g Hospital Infantil de México, Federico Gómez, CDMX, Mexico; h Colorado State University, Fort Collins, CO, USA; i Sant Pau Mental Health Research Group, Institut d’Investigació Biomédica Sant Pau, Hospital de la Santa Creu I Sant Pau, Barcelona, Spain; j Department of Psychiatry and Forensic Medicine, Universitata Autònoma de Barcelona, Barcelona, Spain; k CIBERSAM, Carlos III Health Institute, Madrid, Spain; l Institute on Digital Health and Innovation, Florida State University, Tallahassee, FL, USA; m Department of Family and Community Health, Penn Nursing, University of Pennsylvania, Philadelphia, PA, USA; n Sistema Nacional de Investigación, SENACYT, Panama City, Panama; o Calle Elvira Meléndez, Edificio Towerbank, Piso28, Panama City, Panama; p 534A Calle Guayacanes, Panama City, Panama

**Keywords:** Psychiatric morbidity, Trauma, Mental health conditions, Migrants in transit, Americas, Darién

## Abstract

**Background:**

Migration through the Darién Gap en route to the US peaked in 2023, with hundreds of thousands of people in transit exposed to physical and psychological health hazards. However, systematic data on mental health conditions for this population remain limited. This study explored the prevalence of psychological symptoms, mental disorders, exposure to psychological trauma, and health-related quality of life among migrants in transit through Darién and described age- and sex-based differences.

**Methods:**

In January 2022, we conducted a cross-sectional mental health assessment at a government-run Migrant Reception Station in Darién, Panama. Randomly selected adults and adolescents aged ≥12 years completed the Refugee Health Screener-15 (RHS-15) and the EUROHIS-QoL-8. Those who screened positive were offered clinical evaluation by mental health specialists using the Mini International Neuropsychiatric Interview.

**Results:**

Among 137 participants (median 30.0 years [IQR 11.0]; 54.1 % male), 65.0 % screened positive for psychological distress, and 35.6 % met the diagnostic criteria for at least one psychiatric disorder. Trauma- and stress-related disorders (24.4 %) and depressive disorders (17.0 %) were most prevalent. Females reported higher exposure to psychological trauma during the crossing relative to males (43.6 % vs. 23.3 %; *p* = 0.01) and higher prevalence of history of mental disorder (25.8 % vs. 5.5 %; *p* = 0.001). A larger proportion of adolescents and younger adults [12–24 years] scored at-risk for suicide compared with older participants [ages 25 years and older] (36.7 % vs. 10.5 %, *p* = 0.001). Findings highlight significant mental health burden during transit and suggest the potential utility of targeted screening and referral mechanisms in reception settings.

## Highlights

• This study provides the first systematic, evidence-based, clinically and culturally appropriate quantitative assessment of mental health outcomes among a diverse population of migrants assessed immediately after transiting the Darién Gap.
• We found a high prevalence of diagnosable and subclinical mental health conditions, with women and youth identified as particularly at-risk groups.
• Findings support the integration of mental health and psychosocial support as a standard component of the humanitarian response while in transit.
• The two-stage screening model utilized in this study offers a blueprint for triage in high-volume, resource-limited environments.

## 1 Introduction

Migration of people from around the world through the Darién Gap towards North America has intensified in recent years, driven by overlapping crises of political instability, economic hardship, environmental degradation, and social inequality (United Nations High Commissioner for Refugees, 2025; Ratha et al., 2024; United Nations High Commissioner for Refugees 2024b; Gabster et al., 2021; United Nations High Commissioner for Refugees 2024a). Although some people’s migration is indeed voluntary (for example, to reunify families or to better social status), others’ migration is to escape significant social and political threats—circumstances that leave them with few viable alternatives. The COVID-19 pandemic exacerbated these pressures, further destabilizing livelihoods and disrupting essential health services (Pinzón-Espinosa et al. 2021; Espinel et al. 2020). By 2023, over 120 million people had been displaced worldwide—including 43.4 million refugees, 68.3 million internally displaced persons, and 6.9 million asylum seekers—underscoring the scale of this global humanitarian crisis (United Nations High Commissioner for Refugees 2024a).

The Darién Gap, a dense jungle spanning the Colombia–Panama border that is historically difficult to pass, epitomizes the dangers of transit migration. The Darién Gap has become a significant route toward North America in recent years (United Nations High Commissioner for Refugees 2024b; United Nations High Commisioner for Human Rights, 2016; Lancet 2023; Pan American Health Organization and World Health Organization, 2024; Higuita et al., 2022). Migration records from Panama reveal a staggering almost 1000-fold increase in just over a dozen years: from just 559 irregular crossings in 2010 to over 520,000 in 2023, including 113,180 minors [under age 18 years of age] (Servicio Nacional de Migración, 2025). The number of migrants through the Darién Gap fell to 302,203 in 2024, and then dramatically decreased following January 20, 2025 [inauguration of U.S. President Trump and corresponding changes to U.S. border policies], with 2927 migrants in the first half of 2025 and only 96 of these in the second quarter (April-June) 2025 (Servicio Nacional de Migración, 2025; United Nations High Commissioner for Refugees, 2025). The demographic composition of people in transit through the Darién Gap has been historically dominated by populations from across Latin America; recent flows are led by Venezuelans, Haitians, and Ecuadorians, alongside increasing numbers from Asia and sub-Saharan Africa (Servicio Nacional de Migración, 2025). Extra-continental individuals arrive at a South American country and later travel north. Furthermore, compared to before 2015, when migrants in transit were mostly men, recent years have seen an increase in women and minors among migrants in transit through the Darién Gap, signalling an increase in family migration (Servicio Nacional de Migración, 2025). The large number of minors exposes structural gaps for this population in social protection, paediatric care, and psychosocial support. These population movement patterns underscore the urgent need for integrated public health responses that are attuned to the evolving complexity of migration through the Darién Gap.

Migrants who are in transit to another country face considerable risks to their health and safety. In 2022, more than 7205 people died or went missing on migratory routes globally (International Organization for Migration, n.d.). Overland corridors through the Americas—particularly those leading to the U.S.–Mexico border—are among the deadliest in the world (International Organization for Migration, n.d.; Lancet 2023). Hundreds of deaths were reported in 2024 along key routes, including the Darién Gap, northern Chile, and Mexico’s northern frontier (Pan American Health Organization and World Health Organization, 2024). Insecurity including environmental hazards, sexual and physical violence, and a profound lack of access to food, shelter, and healthcare mark some of the dangers found on these routes (Keller et al. 2017; Edberg et al. 2021; Higuita et al., 2022). Many of the asylum-seeking individuals residing in the United States who journeyed through the Darién Gap experienced hazardous traumatic events including physical assault, sexual assault, unlawful detention, kidnapping, extortion, poor health and psychiatric illnesses (Olivo-Freites et al. 2025). Systematic data on health, especially mental health, among those in transit across the Americas to reach the Mexico-U.S. border remain limited (Pierroz and Santos-Figueroa 2024; Pan American Health Organization, 2024).

Migrants in transit—whom we define as individuals currently undertaking an ongoing journey and not yet at their intended destination, irrespective of formal legal status (e.g., refugee, asylum applicant, undocumented)—constitute a population whose experiences are shaped by the conditions of the travel phase itself. This category is distinct from refugees and asylum seekers as it is based on travel phase, encompasses heterogeneous legal statuses, and captures unique exposures and service needs inherent to the transit period. Migrants in transit may endure intense psychological distress due to legal status, discrimination, and disruption of social ties (Fazel et al. 2012; Porter and Haslam 2005; Lindert et al. 2009; Miller and Rasmussen 2017; Stoesslé et al. 2015). However, little is known about the mental health burden among migrants actively crossing routes like the Darién Gap (Pierroz and Santos-Figueroa 2024; Pan American Health Organization, 2024). Without such data, it remains difficult to design effective care models or targeted interventions that aim to lessen psychological distress while in transit.

To address the limited data available on the mental health needs of migrants in transit, we conducted a rapid epidemiological assessment of mental health conditions at a government-run Migrant Reception Station (MRS) in Darién, situated at the exit of the Darién Gap (Gabster et al., 2021). This study is part of an integrated protocol of multiple health topics; previously, we reported on sexual and reproductive health, healthcare access, and nutrition among migrants in transit (Erausquin et al., 2022; Panchenko et al. 2023; Katz et al. 2022). In the present study, we present findings on mental health conditions from the same cross-sectional sample. The study aims to explore the prevalence of psychological symptoms, mental disorders, trauma exposure, and health-related quality of life among migrants in transit through Darién and to examine variability in these outcomes by sex and age. Our findings can inform context-adapted policies and care systems for migrants in transit and, more broadly, uncover vulnerabilities and suggest new areas for research among migrants in transit worldwide.

## 2 Methods

### 2.1 Study design and setting

This observational cross-sectional study was conducted in January 2022 at a government-run Migrant Reception Station (MRS) near Metetí, Darién, Panama—the first official point of contact for individuals exiting the Darién Gap. The study was part of a larger rapid epidemiological assessment designed to evaluate health needs among migrants in transit through Panama, and the full study protocol was peer-reviewed and published (Gabster et al., 2021). The sample size for the clinical components, including mental health, was determined *a priori* based on prior data on physical health among migrants in transit through the Darién Gap and aimed for *n* = 120 participants under age 18 years and *n* = 160 participants age 18 and older (Gabster et al., 2021). Members of the target population, study participants, and the public were not involved in the design, conduct, reporting, or dissemination plans of this research. The STROBE checklist for this cross-sectional study is available in the online supplementary material.

### 2.2 Participant selection

Participant recruitment procedures have been described in detail in the published protocol (Gabster et al., 2021). In brief, travel groups (2–8 individuals) arriving at the MRS were introduced to the study. From each group, one adult (aged 18 years or older) and one adolescent (aged 12–17 years) were randomly selected to be invited to participate. Consent processes were conducted privately to avoid peer influence. Children <12 years were excluded from the mental health component but were included in other health components.

### 2.3 Research team

The on-site mental health team comprised a clinical psychologist experienced in forced migration and trauma (TR), a licensed mental health nurse (EFM), a psychiatrist with clinical and research expertise in severe mental illness (JPE), and a trained mental health support worker (GCT). All assessments were conducted in the participant's preferred language (Spanish, French, Haitian Creole, Portuguese, or English), with the assistance of a study team interpreter if necessary.

### 2.4 Instruments and procedures

All participants completed the Refugee Health Screener-15 (RHS-15), a validated screening tool for anxiety, depression, somatization, and post-traumatic stress symptoms in displaced populations (Hollifield et al. 2013, 2016). This tool, although initially designed for refugee populations, has been used in migrant populations without formal refugee status in both clinical and research applications to screen for psychological distress (U.S. Centers for Disease Control and Prevention CDC, 2025; Lipscomb 2019). The tool was administered either self-reported or with professional assistance, based on each participant’s preference, using linguistically validated versions (Pathways to Wellness 2020).

Following the RHS-15, participants completed the 8-item version of the WHO Quality of Life Instrument (EUROHIS-QOL-8) to assess health-related quality of life (da Rocha et al., 2012; Bullinger and Quitmann 2014). The EUROHIS-QOL-8 is a cross-culturally validated, brief instrument to capture self-assessed well-being, and it has been used among displaced populations to describe health beyond specific diagnoses and conditions (Buchcik et al. 2023; Gagliardi et al. 2021). Quality of life is non-disease-specific and “captur[es] changes of health that matter to the patients and the societies they live in,” (Bullinger and Quitmann 2014, p. 138.) As with the RHS-15, the tool was administered either self-reported or with professional assistance, based on participant preference. The EUROHIS-QOL-8 is available in Spanish, French, Portuguese, and English (Pires et al. 2018; da Rocha et al., 2012; Schmidt et al. 2006); the Haitian Creole version was professionally translated for our study.

Participants with a positive RHS-15 score (e.g., ≥12 total and/or distress thermometer ≥5) were offered a clinical assessment including a structured diagnostic interview using the Mini International Neuropsychiatric Interview (MINI) conducted by either the psychiatrist (JPE) or the mental health nurse (EFM). The MINI assesses DSM-IV-TR criteria for common psychiatric disorders; translated and validated versions in the study languages were accessed from the publisher (https://harmresearch.org/mini-international-neuropsychiatric-interview-mini/). Clinicians also recorded any clinically observed mental illness outside the MINI’s scope or based on participant-referred previous history of psychiatric disorders. Experience of a traumatic event before or during transit was noted for participants [separate from meeting clinical criteria for diagnosis of acute post-traumatic stress reaction]. Detailed information on how information from screening tools and diagnoses were categorized for analysis is available in the online supplementary material.

Consistent with our published study protocol (Gabster et al., 2021), all participants were offered a brief voluntary counselling session with either the psychologist (TR) or the psychiatrist (JPE), regardless of the screening outcome. The protocol also included guidance for distress, psychosis, and/or risk of violence to self or others, with strategies from verbal containment to pharmacologic management (Gabster et al., 2021).

### 2.5 Statistical analysis

Descriptive statistics were used to summarize participant demographics and screening outcomes. Continuous variables were reported as medians with interquartile ranges. Categorical variables were presented as frequencies and percentages. Associations of psychological symptoms, mental disorders, trauma exposure, and health-related quality of life with sex (male/female) and age group (ages 24 and younger/ages 25 and older) were examined using bivariate analyses, including Mann-Whitney *U tests* for continuous variables and chi-square analysis for categorical variables. All analyses were conducted using Stata 18 (StataCorp, 2023).

### 2.6 Ethical considerations

Study approval was obtained from the Ministry of Health, the National Migration Service, and the National Border Service of Panama. The institutional bioethics committee of Instituto Conmemorativo Gorgas de Estudios de la Salud reviewed and approved the protocol (approval number 260/CBI/ICGES/21). All participants provided written informed consent; for adolescents aged 12–17, written informed assent was obtained alongside written guardian consent. All components of the mental health assessment were voluntary and confidential. As described above, the primary purpose of the mental health assessment was rapid epidemiological assessment. We had procedures in place for managing acute psychiatric problems and participants were offered brief psychological counselling but given the active in-transit status of the population, the study was not intended to directly connect individuals to ongoing care.

## 3 Results

Our analytic sample consisted of a total of 137 individuals who completed the RHS-15 and had valid information on age and sex; 135 of these also had information on the EUROHIS-QoL-8, the MINI, and an assessment by a mental health professional. Seventy-one participants accepted either psychological and/or psychiatric support after undergoing screening assessments. Four participants were actively taking any psychotropic medication (one anxiolytics - benzodiazepines, one SSRI, and one long-acting injectable first-generation antipsychotic). Three were carrying their own medication; one required on-site prescription of medication by the evaluating psychiatrist, a rapid, short-acting benzodiazepine for acute distress relief.

As shown in Table 1, the sample was 54.1 % male, with a median age of 30 years (IQR 11.0). The highest level of education for most participants (47.3 %) was some secondary education. Nearly half (45.1 %) of the sample indicated that their country of origin was Latin America, with an additional 31.5 % from the Caribbean, 19.8 % from Africa, and 3.6 % from Asia. As shown in Table 2, the prevalence of several mental health conditions was high, with 17.0 % of participants meeting DSM-5 clinical criteria for depressive disorders, 10.4 % for one or more anxiety and related disorders, and 24.4 % for trauma- and stressor-related disorders. Psychotic disorders (2.2 %), obsessive-compulsive and related disorders (0.7 %), alcohol use disorder (3.7 %), and substance use disorder (2.2 %) had lower prevalence. We found 35.6 % of the sample met criteria for mental health diagnosis (17.8 % one diagnosis and 17.8 % two or more diagnoses).

**Table 1:** Demographic characteristics of study participants (migrants in transit, age ≥12 years) by sex and age, ERM January 2022.

	Total Sample		Female		Male		p-value	Younger		Older		p-value
								*Age 12-24y*		*Age 25-65y*		
	Freq.	(%)	Freq.	(%)	Freq.	(%)		Freq.	(%)	Freq.	(%)	
**Sex**												0.926
Female	62/135	(45.9)	–		–			14/30	(46.7)	48/105	(45.7)	
Male	73/135	(54.1)	–		–			16/30	(53.3)	57/105	(54.3)	
**Age**							0.996					
12-17	8/135	(5.9)	4/62	(6.5)	4/73	(5.5)		–		–		
18-24	22/135	(16.3)	10/62	(16.1)	12/73	(16.4)		–		–		
25-39	77/135	(57.1)	35/62	(56.5)	42/73	(57.5)		–		–		
40 or older	28/135	(20.7)	13/62	(21.0)	15/73	(20.6)		–		–		
**Education**							0.930					0.512
Up to Grade 6 / primary school	14/110	(12.7)	6/51	(11.8)	8/59	(13.6)		1/20	(5.0)	13/90	(14.4)	
Grades 7-12 / secondary school	52/110	(47.3)	25/51	(49.0)	27/59	(45.8)		10/20	(50.0)	42/90	(46.7)	
Beyond secondary school	44/110	(40.0)	20/51	(39.2)	24/59	(40.7)		9/20	(45.0)	35/90	(38.9)	
**Region of Origin**							**<0.001**					0.078
Caribbean	35/111	(31.5)	28/51	(54.9)	7/60	(11.7)		3/20	(15.0)	32/91	(35.2)	
Latin America	50/111	(45.1)	17/51	(33.3)	33/60	(55.0)		14/20	(70.0)	36/91	(39.6)	
Asia	4/111	(3.6)	0/51	(0.0)	4/60	(6.7)		1/20	(5.0)	3/91	(3.3)	
Africa	22/111	(19.8)	6/51	(11.8)	16/60	(26.7)		2/20	(10.0)	20/91	(22.0)	

Continuous Variables	Total Sample		Female		Male			Younger		Older		
	Median	IQR	Median	IQR	Median	IQR	p-value	Median	IQR	Median	IQR	p-value
**Age (range: 14-65 years)**	30	11	29	8.5	31	13	0.27	20	5	32	11	**<0.001**

Note: Sample size is 135 for all analyses except education (n=110) and region of origin (n=111) due to nonresponse for those self-report items. P-values are the values associated with the appropriate tests of difference by sex (female or male), and by age group (24 years or younger, 25 years or older). Mann-Whitney u-tests were used for continuous variables and chi square analyses were used for categorical variables.

**Table 2:** Psychological characteristics of study participants (migrants in transit, age ≥12 years) by sex and age, ERM January 2022.

	Total Sample		Female		Male		p-value	Younger		Older		p-value
								*Age 12-24y*		*Age 25-65y*		
	Freq.	(%)	Freq.	(%)	Freq.	(%)		Freq.	(%)	Freq.	(%)	
**Psychotic Disorders**	3/135	(2.2)	2/62	(3.2)	1/73	(1.4)	0.47	1/30	(3.3)	2/105	(1.9)	0.64
**Mood Disorders**	23/135	(17.0)	12/62	(19.4)	11/73	(15.1)	0.51	7/30	(23.3)	16/105	(15.2)	0.30
**Anxiety Disorders**	14/135	(10.4)	5/62	(8.1)	9/73	(12.3)	0.42	5/30	(16.7)	9/105	(8.6)	0.20
**Obsessive-Compulsive Disorder**	1/135	(0.7)	0/62	(0.0)	1/73	(1.4)	0.36	1/30	(3.3)	0/105	(0.0)	0.06
**Trauma- and stress-related Disorders**	33/135	(24.4)	22/62	(35.5)	11/73	(15.1)	**0.01**	9/30	(30.0)	24/105	(22.9)	0.42
**Alcohol Use Disorder**	5/135	(3.7)	2/62	(3.2)	3/73	(4.1)	0.79	2/30	(6.7)	3/105	(2.9)	0.33
**Substance Use Disorder**	3/135	(2.2)	2/62	(3.2)	1/73	(1.4)	0.47	2/30	(6.7)	1/105	(1.0)	0.06
**At-risk for Suicide**	22/135	(16.3)	13/135	(21.0)	9/73	(12.3)	0.18	11/30	(36.7)	11/105	(10.5)	**0.001**
**Suicide Risk Level**							0.42					0.82
Low	17/22	(77.3)	9/13	(69.2)	8/9	(88.9)		9/11	(81.8)	8/11	(72.7)	
Medium	3/22	(13.6)	2/13	(15.4)	1/9	(11.1)		1/11	(9.1)	2/11	(18.2)	
**High**	2/22	(9.1)	2/13	(15.4)	0/9	(0.0)		1/11	(9.1)	1/11	(9.1)	
**RHS Screen Positive**	89/137	(65.0)	47/64	(73.4)	42/73	(57.5)	0.05	20/30	(64.5)	69/106	(65.1)	0.95
**MH Diagnoses**							0.36					0.10
None	87/135	(64.4)	36/62	(58.1)	51/73	(69.9)		15/30	(50.0)	74/105	(70.5)	
One	24/135	(17.8)	13/62	(21.0)	11/73	(15.1)		8/30	(26.7)	14/105	(13.3)	
Two or More	24/135	(17.8)	13/62	(21.0)	11/73	(15.1)		7/30	(23.3)	17/105	(16.2)	
**History of MH Diagnoses**	20/135	(14.8)	16/62	(25.8)	4/73	(5.5)	**0.001**	7/30	(23.3)	13/105	(12.4)	0.14
**History of MH care**	24/135	(17.8)	15/62	(24.2)	9/73	(12.3)	0.07	6/30	(20.0)	18/105	(17.1)	0.72
**Trauma Experienced Prior to Crossing**	32/135	(23.7)	14/62	(22.6)	18/73	(24.7)	0.78	12/30	(40.0)	20/105	(19.1)	**0.02**
**Trauma Experienced During Crossing**	44/135	(32.6)	27/62	(43.6)	17/73	(23.3)	**0.01**	12/30	(40.0)	32/105	(30.5)	0.33
**Trauma Witnessed During Crossing**	6/135	(4.4)	4/62	(6.5)	2/73	(2.7)	0.30	1/30	(3.3)	5/105	(4.8)	0.74

Continuous Variables	Total Sample		Female		Male			Younger		Older		
	Median	IQR	Median	IQR	Median	IQR	p-value	Median	IQR	Median	IQR	p-value
**Quality of Life** (range: 1.63-4.5)	3.5	0.75	3.38	0.63	3.63	0.75	0.34	3.38	1.00	3.5	0.63	0.23
**RHS-15 Score** (range: 0-50)	13.0	13.0	15.0	14.5	9	12.0	**0.001**	15.0	16.0	12.0	13.0	**0.04**

Note: Sample size is 135 for MINI and mental health diagnoses; 137 for RHS-15; 128 for Quality of Life. Mental health conditions were not mutually exclusive, and participants could be diagnosed with more than one. Self-reports of experience of or witness to traumatic events were assessed separately from meeting clinical criteria for trauma- and stress-related disorders. P-values are the values associated with the appropriate tests of difference by sex (female or male), and by age group (24 years or younger, 25 years or older); p-values less than 0.05 in bold. Mann-Whitney u-tests were used for continuous variables and chi square analyses were used for categorical variables.

Nearly one in six (16.3 %) of the sample met the threshold for suicide risk; of these, the majority were at low risk for suicide (77.3 %). The RHS-15 had a Cronbach’s alpha of 0.8805 in this study sample. Nearly two-thirds (65.0 %) screened positive on the RHS-15, and the median RHS-15 score was 13.0 (IQR 13.0). Nearly one in five (17.8 %) of the sample had prior interactions with mental health care providers, and 14.8 % had a prior mental health diagnosis in their lifetime. While nearly one-fourth (23.7 %) had experienced a traumatic event prior to crossing the Darién Gap, one-third (32.6 %) experienced a traumatic event during the crossing. The EUROHIS-QOL-8 had a Cronbach’s alpha of 0.6918 in this study sample. Quality of life measured using this instrument was moderate, with a median of 3.5 (IQR 0.75; range 1.63–4.5).

Bivariate analyses by sex (male/female) showed few differences in demographic characteristics, except region of origin. Most males were from Latin America (55.0 %) or Africa (36.7 %), while most females were from the Caribbean (54.9 %) or Latin America (33.3 %). Females showed a higher prevalence of trauma and related disorders (35.5 %) compared to males (15.1 %), *p* = 0.006. A higher proportion of females screened positive on the RHS-15 (73.4 % compared to 57.5 % for males, approaching statistical significance at *p* = 0.052), and similarly, their RHS-15 median score was higher (15.0 compared to 9.0 for males, *p* = 0.001). Females were also more likely than men to report prior mental health diagnoses (25.8 % vs. 5.5 %, *p* = 0.001) and the results for receiving prior mental health care showed a similar pattern and approached statistical significance (24.2 % vs. 12.3 %, *p* = 0.072). A significantly higher proportion of females reported experiencing a traumatic event during the Darién crossing (43.6 % for females and 23.3 % for males, *p* = 0.012).

Bivariate analyses by age (comparing participants aged 12–24 with those ages 25 or older) also showed some important differences. For demographic characteristics, chi-squared analysis of differences by age group in region of origin approached statistical significance (*p* = 0.078), with most younger participants from Latin America (70.0 %). In comparison, older participants were nearly evenly split between Latin American (39.6 %) and the Caribbean (35.2 %). There were no significant differences by age in currently meeting specific mental health diagnostic criteria, though there was a trend such that more younger participants had one (26.7 %) or two or more (23.3 %) diagnoses, compared with older participants (13.3 % and 16.2 %, respectively, *p* = 0.095). Though there were not notable differences in the *proportion that screened positive* on the RHS-15, the RHS-15 median score was higher among younger participants (15.0) compared with older participants (12.0; *p* = 0.09). Comparing participants aged 12–24 with those ages 25 or older, a greater proportion of the younger participants were at risk for suicide compared to the older participants (36.7 % compared with 10.5 %, *p* = 0.001). Similarly, more younger participants experienced a traumatic event in their lifetime prior to crossing (40.0 %) compared with older participants (19.1 %, *p* = 0.017).

Within each sex group, females and males, we also tested for differences by age group in demographic characteristics, mental health, and experiences (results not shown). Among females, there were significant age differences for traumatic events prior to their Darién crossing, with 50.0 % of younger females but only 14.6 % of older females reporting prior traumatic events (*p* = 0.005). Among males, there were significant age differences in being at risk for suicide, with 37.5 % of younger males but only 5.3 % of older males being at risk for suicide (*p* = 0.001). There was also a trend approaching statistical significance whereby younger males had a higher prevalence of meeting diagnostic criteria for anxiety disorders (25.0 % compared with older males at 8.8 %; *p* = 0.081).

### 4 Discussion

To the best of our knowledge, our study represents one of the first quantitative, structured, clinical assessments of mental health among a diverse sample of migrants in transit, immediately after emerging from the Darién Gap. Although our assessment was conducted in early 2022, subsequent migration peaks in 2023 further underscored the urgency of understanding the mental health burden of people in transit through the Darién Gap. Recent policy shifts in 2025 have temporarily reduced transit through the Darién Gap, but the humanitarian crisis of migration in the Americas persists along multiple routes, highlighting the continuing relevance of our findings.

We found that nearly two thirds of our sample screened positive for psychological distress, and more than a third met clinical criteria for a psychiatric disorder—mainly trauma-related and mood disorders. Approximately one in 8 participants reported suicide risk; moreover, adolescents (37 %) and women with a previous history of mental disorder (26 %) were two particularly affected subgroups. One in three participants reported traumatic experiences during the Darién Gap crossing. Female and younger participants had the highest psychological burden, as they showed significantly higher distress scores, rates of traumatic experiences, and previous history of mental disorders. Notably, despite the psychological distress, mental disorder rates, and traumatic experiences, self-reported perceived quality of life remained moderately high across the sample and most subgroups.

There is little evidence in the published literature on the mental health and quality of life assessment of actively migrating populations. However, some parallels may be drawn between this population of migrants exposed to traumatic experiences and violence during transit and other populations. Our findings are consistent with previous reports of psychiatric morbidity among populations exposed to severe trauma: WHO estimates the prevalence of mental disorders in conflict-affected populations to be 22.1 % at any point in time (Charlson et al. 2019). WHO and PAHO have already identified the Darién Gap as a high-risk setting for cumulative trauma with exposure to life-threatening conditions like violence, detention, and lack of access to basic and healthcare services (Pan American Health Organization, 2024; World Health Organization, 2025). Previous studies have emphasized the importance of considering the full migration journey in mental health frameworks, including pre-migration experience of trauma, in-transit exposures, and post-migration stressors (Porter and Haslam 2005; Miller and Rasmussen 2017).

Sex- and age-related differences in our sample reflect previously reported vulnerabilities (Lindert et al. 2009). Women reported significantly higher rates of psychological distress and traumatic events experienced during crossing when compared to male participants, and were also more likely to have a previous psychiatric diagnosis. Women and girls migrating through the region have been reported to be disproportionately exposed to gender-based violence, insecurity, and family separation (Cortes, 2022). Studies in other regions, including the Middle East and Central Asia, have shown that migrant women face disproportionate psychological distress compared with men (Handiso et al. 2024; Ayika et al. 2018). Furthermore, we found a high prevalence of psychological distress and suicide risk among younger migrants in transit. Again, we look at evidence from populations similar to our sample of adolescent and adult migrants in transit: suicidality has been previously reported to be higher in refugees compared to people living in host countries, and male refugees have been reported to display higher suicidal ideation prevalence than female refugees (Bevione et al. 2024). In addition, among migrant children and adolescents who were resettled in high-income countries, rates of being at-risk for suicide were similar to our findings (Fazel et al. 2012; van der Boor et al., 2020). Interestingly, the moderate self-rated quality of life scores our study contrasted with the overall apparent burden of symptoms and the contextual situation. Though counterintuitive, this has been noted in other settings as a “resilience paradox,” where displaced people report hope and resilience despite psychological distress, particularly in the context of forward movement or recent survival (Bonanno 2021; Bonanno et al. 2024; Blakeney 2025; Nickerson et al. 2024). Cultural framing, spiritual coping, goal orientation, among other mechanisms may be responsible for the mediation of these responses (Bonanno 2021; Bonanno et al. 2024; Blakeney 2025; Nickerson et al. 2024).

The high burden of psychological distress, high prevalence of diagnosable psychiatric disorders, and considerable suicide risk in this actively transiting population underscores the need to address the current gaps in healthcare access, including mental health. Although recent policy changes have temporarily reduced migration flows through the Darién Gap, the risks of trauma and psychological distress identified in our study remain highly relevant. Similar vulnerabilities are likely to be present among migrants in other transit corridors worldwide, underscoring the broader applicability of these findings. Integration of services into border reception centres is feasible and increasingly urgent. Long-term consequences of stress and trauma endured by migrants in transit likely produce emotional scars that affect their quality of life and well-being during their resettlement process.

### 4.1 Strengths and limitations

Several strengths of our study underscore the relevance of our findings. First, we achieved high participation and a diverse sample despite working with a population in active transit within a remote and difficult-to-access region of Panama. Second, adherence to a previously published protocol coupled with the use of standardized instruments by clinical experts within a multidisciplinary, multilingual, multicultural team allowed for a robust assessment of a highly diverse population (Gabster et al., 2021). Our sample reflected a globally diverse migrant population, with individuals originating from different parts of Latin America, the Caribbean, Africa, and Asia. Notably, the mental health component was conducted in parallel and in close collaboration with other health assessments, including sexual and reproductive health, nutrition and general medicine, conducted by colleagues from the same integrated research effort (Gabster et al., 2021; Erausquin et al., 2022). This on-site coordination fostered whole-person, trauma-informed care and ensured culturally appropriate engagement with migrants in transit. Nonetheless, our study has several limitations. First, some clinical interviews were necessarily brief due to the urgency of participants’ onward migration, and all were limited by the unavailability of sources to verify previously made diagnoses, medications, or conditions. Second, due to the absence of specialists in child mental health, study participants under 12 years of age were not included in the mental health component. Finally, the cross-sectional design precludes the inferring of causal relationships.

## 5 Conclusion

Migrants crossing the Darién Gap confront extreme adversity, reflected in high rates of psychological distress, trauma, and suicide risk. Women and young people are disproportionately affected, underscoring that migration is not only a political or logistical challenge but also a profound public health issue. Our findings provide further evidence on the need to integrate mental health and psychosocial support as a standard component of the humanitarian response while in transit, and the two-stage screening process utilized in data collection offers a blueprint for triage in high-volume, resource-limited environments. Key areas for future research include the study of long-term mental health trajectories, the mechanisms of psychological resilience, and the need for development of assessment tools and interventions for children under 12.

To address the critical needs of migrants in transit, embedding mental health support into humanitarian infrastructures is essential, and this includes dedicating resources and developing mobile, flexible services that can accompany migrants along their journey. Such measures are critical to protect the dignity, resilience, and well-being of people on the move.

## Funding information

This project was supported by funds and donations of supplies from Instituto Conmemorativo Gorgas de Estudios de la Salud and Community Development Network of the Americas. Dr. Erausquin’s contributions to this publication were supported by the National Institute of Mental Health of the National Institutes of Health under award number R15MH129191. The funders had no role in the design and conduct of the study; collection, management, analysis, and interpretation of the data; preparation, review, or approval of the manuscript; and decision to submit the manuscript for publication. The content is solely the responsibility of the authors and does not necessarily reflect the official views of the funders.

## Data sharing statement

Deidentified individual participant data and the data dictionary that support the findings of this study are available from the corresponding author upon reasonable request, following approval of a methodologically sound proposal and execution of a data access agreement. Data will be available for researchers whose proposed use has been approved for purposes of replicating results or pursuing related research questions.

## CRediT authorship contribution statement

**Justo Pinzón-Espinosa:** Writing – review & editing, Writing – original draft, Project administration, Investigation, Formal analysis, Data curation, Conceptualization. **Jennifer Toller Erausquin:** Writing – review & editing, Writing – original draft, Methodology, Investigation, Formal analysis, Data curation, Conceptualization. **Eugenia Flores Millender:** Writing – review & editing, Supervision, Conceptualization. **Tomás Rincón:** Writing – review & editing, Investigation. **Gonzalo Cabezas Talavero:** Writing – review & editing, Investigation. **Carlos Franco-Paredes:** Writing – review & editing. **Narcis Cardoner:** Writing – review & editing. **Casey D. Xavier Hall:** Writing – review & editing. **Frankie Y. Wong:** Writing – review & editing. **Liying Wang:** Writing – review & editing. **Carmen Alvarez:** Writing – review & editing. **Amanda Gabster:** Writing – review & editing, Writing – original draft, Resources, Project administration, Funding acquisition, Conceptualization.

## Declaration of competing interest

The authors declare the following financial interests/personal relationshipswhich may be considered as potential competing interests: Dr. Pinzón-Espinosa reported CME speaker fees and other nonfinancial honoraria from Otsuka, Idorsia, Neuraxpharm, Casen, Janssen, Angelini Pharma, and Lundbeck, unrelated to the current work. The authors have no other competing interests to declare.

## Appendix Supplementary materials

Image, application 1

Image, application 2

Image, application 3
