# Supplementary material for: Mental health and quality of life of migrants in transit through the Darién gap: A cross-sectional assessment in Panama
Source: J Migr Health. 2026 Mar 20;13:100405. doi: 10.1016/j.jmh.2026.100405 (PMC13081703; doi:10.1016/j.jmh.2026.100405)
Supplement: Supplementary file 1 [file mmc1.docx]

# **Supplement on Diagnostic Categories**

Mental disorder diagnoses, both derived from the MINI International Neuropsychiatric Interview^1^ modules and mental health specialist clinical interviews, were grouped into broader categories to facilitate quantitative analyses with the limited sample size. Supplementary Table 1 shows the expert-consensus broad diagnostic categories, and the diagnoses included in each. ICD-10 codes refer to World Health Organization International Classification of Diseases 10^th^ Edition.^2^

## **Supplementary Table 1. Broad diagnostic categories and included diagnoses in each with corresponding ICD-10 codes.**

 * These are not a part of the MINI but were also clinically assessed on the field.

| Broad Diagnostic Categories | Included diagnoses (timeframe assessed) | ICD-10 codes |
| --- | --- | --- |
| Depressive disorders | Major Depressive Episode (current, 2 weeks)  Recurrent Major Depressive Disorder | F32.x |
|  | Major Depressive Episode with melancholic symptoms (current, 2 weeks) | F33.x |
|  | Persistent depressive disorder (Dysthymia) (current, 2 years) | F34.1 |
| Bipolar and related disorders | Manic episode (current or past) | F30.x – F31.9 |
|  | Hypomanic episode (current or past) | F31.8-F31.9/F34.0 |
| Anxiety disorders | Panic disorder (current or lifetime) | F40.01-F41.0 |
|  | Agoraphobia (current) | F40.0 |
|  | Social anxiety disorder (Social phobia) (last month) | F40.1 |
|  | Generalized Anxiety Disorder (current, prior 6 months) | F41.1 |
| Schizophrenia-spectrum disorders | Psychotic Disorders (current or lifetime) | F20.x-F29 |
|  | Mood disorder with psychotic features (current) | F32.3/F33.3 |
| Obsessive-Compulsive and related disorders | Obsessive-Compulsive Disorder | F42.8 |
| Trauma- and stressor-related disorders | Acute post-traumatic stress reaction (current, last month) | F43.1 |
|  | Borderline personality disorder* | F60.3 |
| Alcohol use disorder | Alcohol Dependence (last 12 months) | F10.2x |
|  | Alcohol Abuse (last 12 months) | F10.1 |
| Substance use disorder | Substance Dependence (last 12 months) | F11.1-F19.1 |
|  | Substance Abuse (last 12 months) | F11.1-F19.1 |
| Suicide risk | Low, Moderate, or High risk (last month) | No specific coding. |
| Eating disorders | Anorexia nervosa (current, last 3 months) | F50.0 |
|  | Bulimia nervosa (current, last 3 months) | F50.2 |
| Antisocial personality Disorder | Lifetime | F60.2 |
| Attention-Deficit/Hyperactivity Disorder* | Assessed clinically for lifetime diagnosis. | F90.x |

# **References**

1 Sheehan DV, Lecrubier Y, Sheehan KH, *et al.* The Mini-International Neuropsychiatric Interview (M.I.N.I.): the development and validation of a structured diagnostic psychiatric interview for DSM-IV and ICD-10. *J Clin Psychiatry* 1998; **59 Suppl 20**: 22-33;quiz 34-57.

2 World Health Organization. International Classification of Diseases (ICD-10). 2010. https://icd.who.int/browse10/2010/en (accessed April 21, 2020).
