# Supplementary material for: Mental health and quality of life of migrants in transit through the Darién gap: A cross-sectional assessment in Panama
Source: J Migr Health. 2026 Mar 20;13:100405. doi: 10.1016/j.jmh.2026.100405 (PMC13081703; doi:10.1016/j.jmh.2026.100405)
Supplement: Supplementary file 3 [file mmc3.docx]

**Tables with 95% CIs**

**Table 1.** Demographic characteristics of study participants (migrants in transit, age ≥12 years) by sex and age, ERM January 2022.

|  | **Total Sample**  Freq. (%) | | **Female**  Freq. (%) | | **Male**  Freq. (%) | | **p-value** | **Younger**  Age 14-24y  Freq. (%) | | **Older**  Age 25-65y  Freq. (%) | | **p-value** |
| --- | --- | --- | --- | --- | --- | --- | --- | --- | --- | --- | --- | --- |
|  | [95%CI] | |  | |  | |  |  | |  | |  |
| **Sex** |  |  |  |  |  |  |  |  |  |  |  | 0.926 |
| Female | 62/135 | (45.9)  [37.6-54.4] | -- |  | -- |  |  | 14/30 | (46.7) | 48/105 | (45.7) |  |
| Male | 73/135 | (54.1)  [45.6-62.4] | -- |  | -- |  |  | 16/30 | (53.3) | 57/105 | (54.3) |  |
|  |  |  |  |  |  |  |  |  |  |  |  |  |
| **Age** |  |  |  |  |  |  | 0.996 |  |  |  |  |  |
| 12-17 | 8/135 | (5.9)  [3.0-11.5] | 4/62 | (6.5) | 4/73 | (5.5) |  | -- |  | -- |  |  |
| 18-24 | 22/135 | (16.3)  [10.9-23.6] | 10/62 | (16.1) | 12/73 | (16.4) |  | -- |  | -- |  |  |
| 25-39 | 77/135 | (57.1)  [48.4-65.2] | 35/62 | (56.5) | 42/73 | (57.5) |  | -- |  | -- |  |  |
| 40 or older | 28/135 | (20.7)  [14.7-28.5] | 13/62 | (21.0) | 15/73 | (20.6) |  | -- |  | -- |  |  |
|  |  |  |  |  |  |  |  |  |  |  |  |  |
| **Education** |  |  |  |  |  |  | 0.930 |  |  |  |  | 0.512 |
| Up to Grade 6 / primary school | 14/110 | (12.7)  [7.6-20.4] | 6/51 | (11.8) | 8/59 | (13.6) |  | 1/20 | (5.0) | 13/90 | (14.4) |  |
| Grades 7-12 / secondary school | 52/110 | (47.3)  [38.0-56.7] | 25/51 | (49.0) | 27/59 | (45.8) |  | 10/20 | (50.0) | 42/90 | (46.7) |  |
| Beyond secondary school | 44/110 | (40.0)  [31.1-49.5] | 20/51 | (39.2) | 24/59 | (40.7) |  | 9/20 | (45.0) | 35/90 | (38.9) |  |
| **Region of Origin** |  |  |  |  |  |  | **<0.001** |  |  |  |  | 0.078 |
| Caribbean | 35/111 | (31.5)  [23.5-40.8] | 28/51 | (54.9) | 7/60 | (11.7) |  | 3/20 | (15.0) | 32/91 | (35.2) |  |
| Latin America | 50/111 | (45.1)  [36.0-54.5] | 17/51 | (33.3) | 33/60 | (55.0) |  | 14/20 | (70.0) | 36/91 | (39.6) |  |
| Asia | 4/111 | (3.6)  [1.3-9.3] | 0/51 | (0.0) | 4/60 | (6.7) |  | 1/20 | (5.0) | 3/91 | (3.3) |  |
| Africa | 22/111 | (19.8)  [13.4-28.4] | 6/51 | (11.8) | 16/60 | (26.7) |  | 2/20 | (10.0) | 20/91 | (22.0) |  |
|  |  |  |  |  |  |  |  |  |  |  |  |  |
| **Continuous Variables** | **Total Sample** | | **Female** | | **Male** | |  | **Younger** | | **Older** | |  |
|  | **Median** | **IQR** | **Median** | **IQR** | **Median** | **IQR** | p-value | **Median** | **IQR** | **Median** | **IQR** | p-value |
| **Age (range: 14-65 years)** | 30 | 11 | 29 | 8.5 | 31 | 13 | 0.27 | 20 | 5 | 32 | 11 | **<0**.**001** |

Note: Sample size is 135 for all analyses except education (n=110) and region of origin (n=111) due to nonresponse for those self-report items. P-values are the values associated with the appropriate tests of difference by sex (female or male), and by age group (24 years or younger, 25 years or older). Mann-Whitney u-tests were used for continuous variables and chi square analyses were used for categorical variables.

**Table 2.** Psychological characteristics of study participants (migrants in transit, age ≥12 years) by sex and age, ERM January 2022.

|  | **Total Sample**  Freq. (%) | | **Female**  Freq. (%) | | **Male**  Freq. (%) | | **p-value** | **Younger**  Age 12-24y  Freq. (%) | | **Older**  Age 25-65y  Freq. (%) | | **p-value** |
| --- | --- | --- | --- | --- | --- | --- | --- | --- | --- | --- | --- | --- |
|  | [95%CI] | |  | |  | |  |  | |  | |  |
| **Psychotic Disorders** | 3/135 | (2.2)  [0.7-6.7] | 2/62 | (3.2) | 1/73 | (1.4) | 0.47 | 1/30 | (3.3) | 2/105 | (1.9) | 0.64 |
|  |  |  |  |  |  |  |  |  |  |  |  |  |
| **Mood Disorders** | 23/135 | (17.0)  [11.5-24.4] | 12/62 | (19.4) | 11/73 | (15.1) | 0.51 | 7/30 | (23.3) | 16/105 | (15.2) | 0.30 |
|  |  |  |  |  |  |  |  |  |  |  |  |  |
| **Anxiety Disorders** | 14/135 | (10.4)  [6.2-16.8] | 5/62 | (8.1) | 9/73 | (12.3) | 0.42 | 5/30 | (16.7) | 9/105 | (8.6) | 0.20 |
|  |  |  |  |  |  |  |  |  |  |  |  |  |
| **Obsessive-Compulsive Disorder** | 1/135 | (0.7)  [0.10-5.2] | 0/62 | (0.0) | 1/73 | (1.4) | 0.36 | 1/30 | (3.3) | 0/105 | (0.0) | 0.06 |
| **Trauma- and stress-related Disorders** | 33/135 | (24.4)  [17.9-32.5] | 22/62 | (35.5) | 11/73 | (15.1) | **0**.**01** | 9/30 | (30.0) | 24/105 | (22.9) | 0.42 |
| **Alcohol Use Disorder** | 5/135 | (3.7)  [1.5-8.6] | 2/62 | (3.2) | 3/73 | (4.1) | 0.79 | 2/30 | (6.7) | 3/105 | (2.9) | 0.33 |
|  |  |  |  |  |  |  |  |  |  |  |  |  |
| **Substance Use Disorder** | 3/135 | (2.2)  [0.7-6.7] | 2/62 | (3.2) | 1/73 | (1.4) | 0.47 | 2/30 | (6.7) | 1/105 | (1.0) | 0.06 |
| **At-risk for Suicide** | 22/135 | (16.3)  [10.9-23.6] | 13/135 | (21.0) | 9/73 | (12.3) | 0.18 | 11/30 | (36.7) | 11/105 | (10.5) | **0**.**001** |
|  |  |  |  |  |  |  |  |  |  |  |  |  |
| **Suicide Risk Level** |  |  |  |  |  |  | 0.42 |  |  |  |  | 0.82 |
| Low | 17/22 | (77.3) | 9/13 | (69.2) | 8/9 | (88.9) |  | 9/11 | (81.8) | 8/11 | (72.7) |  |
| Medium | 3/22 | (13.6) | 2/13 | (15.4) | 1/9 | (11.1) |  | 1/11 | (9.1) | 2/11 | (18.2) |  |
| **High** | 2/22 | (9.1) | 2/13 | (15.4) | 0/9 | (0.0) |  | 1/11 | (9.1) | 1/11 | (9.1) |  |
| **RHS Screen Positive** | 89/137 | (65.0) | 47/64 | (73.4) | 42/73 | (57.5) | 0.05 | 20/30 | (64.5) | 69/106 | (65.1) | 0.95 |
|  |  |  |  |  |  |  |  |  |  |  |  |  |
| **MH Diagnoses** |  |  |  |  |  |  | 0.36 |  |  |  |  | 0.10 |
| None | 87/135 | (64.4)  [55.9-72.1] | 36/62 | (58.1) | 51/73 | (69.9) |  | 15/30 | (50.0) | 74/105 | (70.5) |  |
| One | 24/135 | (17.8)  [12.2-25.2] | 13/62 | (21.0) | 11/73 | (15.1) |  | 8/30 | (26.7) | 14/105 | (13.3) |  |
| Two or More | 24/135 | (17.8)  [12.2-25.2] | 13/62 | (21.0) | 11/73 | (15.1) |  | 7/30 | (23.3) | 17/105 | (16.2) |  |
|  |  |  |  |  |  |  |  |  |  |  |  |  |
| **History of MH Diagnoses** | 20/135 | (14.8)  [9.7-21.9] | 16/62 | (25.8) | 4/73 | (5.5) | **0**.**001** | 7/30 | (23.3) | 13/105 | (12.4) | 0.14 |
|  |  |  |  |  |  |  |  |  |  |  |  |  |
| **History of MH care** | 24/135 | (17.8)  [12.2-25.2] | 15/62 | (24.2) | 9/73 | (12.3) | 0.07 | 6/30 | (20.0) | 18/105 | (17.1) | 0.72 |
|  |  |  |  |  |  |  |  |  |  |  |  |  |
| **Trauma Experienced Prior to Crossing** | 32/135 | (23.7)  [17.9-32.5] | 14/62 | (22.6) | 18/73 | (24.7) | 0.78 | 12/30 | (40.0) | 20/105 | (19.1) | **0**.**02** |
|  |  |  |  |  |  |  |  |  |  |  |  |  |
| **Trauma Experienced During Crossing** | 44/135 | (32.6)  [25.2-41.0] | 27/62 | (43.6) | 17/73 | (23.3) | **0**.**01** | 12/30 | (40.0) | 32/105 | (30.5) | 0.33 |
|  |  |  |  |  |  |  |  |  |  |  |  |  |
| **Trauma Witnessed During Crossing** | 6/135 | (4.4)  [2.0-9.6] | 4/62 | (6.5) | 2/73 | (2.7) | 0.30 | 1/30 | (3.3) | 5/105 | (4.8) | 0.74 |
|  |  | |  | |  | |  |  | |  | |  |
| **Continuous Variables** | **Total Sample** | | **Female** | | **Male** | |  | **Younger** | | **Older** | |  |
|  | **Median** | **IQR** | **Median** | **IQR** | **Median** | **IQR** | p-value | **Median** | **IQR** | **Median** | **IQR** | p-value |
| **Quality of Life**  (range: 1.63-4.5) | 3.5 | 0.75 | 3.38 | 0.63 | 3.63 | 0.75 | 0.34 | 3.38 | 1.00 | 3.5 | 0.63 | 0.23 |
|  |  |  |  |  |  |  |  |  |  |  |  |  |
| **RHS-15 Score**  (range: 0-50) | 13.0 | 13.0 | 15.0 | 14.5 | 9 | 12.0 | **0**.**001** | 15.0 | 16.0 | 12.0 | 13.0 | **0**.**04** |

Note: Sample size is 135 for MINI and mental health diagnoses; 137 for RHS-15; 128 for Quality of Life. Mental health conditions were not mutually exclusive, and participants could be diagnosed with more than one. Self-reports of experience of or witness to traumatic events were assessed separately from meeting clinical criteria for trauma- and stress-related disorders. P-values are the values associated with the appropriate tests of difference by sex (female or male), and by age group (24 years or younger, 25 years or older); p-values less than 0.05 in bold. Mann-Whitney u-tests were used for continuous variables and chi square analyses were used for categorical variables.
